# Association between extreme temperature exposure and COPD health outcomes in China: study protocol for a systematic review

**DOI:** 10.3389/fmed.2026.1873054

**Published:** 2026-06-25

**Authors:** Jiaxin Xu, Peng Zhang, Yan Wang, Jianya Yang, Suyun Li

**Affiliations:** 1 Department of Respiratory Diseases, The First Affiliated Hospital of Henan University of Chinese Medicine, Zhengzhou, Henan, China; 2 Collaborative Innovation Center for Chinese Medicine and Respiratory Diseases Co-Construction by Henan Province & Education Ministry of P. R. China, Henan University of Chinese Medicine, Zhengzhou, Henan, China; 3 Henan Key Laboratory of Chinese Medicine for Respiratory Disease, Henan University of Chinese Medicine, Zhengzhou, Henan, China; 4 Henan International Joint Laboratory of Evidence-based Evaluation for Respiratory Diseases, Henan Province Clinical Research Center for Respiratory Diseases, The First Affiliated Hospital of Henan University of Chinese Medicine, Zhengzhou, Henan, China

**Keywords:** climate change, COPD, extreme temperature, heat and cold exposure, meta-analysis, systematic review

## Abstract

**Introduction:**

The Chinese continent is grappling with a rising prevalence of chronic obstructive pulmonary disease (COPD) in conjunction with the growing threat of climate change and its associated extreme temperature. This systematic review aims to assess the relationship between extreme temperature exposures (heat and cold exposure) and COPD health outcomes in Chinese populations.

**Methods and analysis:**

This study will assess the relationship between extreme temperature exposures and COPD health outcomes through a systematic review and meta-analysis. Observational studies meeting the inclusion criteria will be included, and the Population-Exposure-Comparator-Outcome-Study (PECOS) research design standard will be adopted to examine how extreme temperatures impact COPD-related mortality and morbidity including hospitalizations and acute exacerbations. A literature search will be conducted across multiple databases, including PubMed, Web of Science, Embase, Chinese National Knowledge Infrastructure (CNKI), WANGFANG Data, VIP and Sinomed. We will evaluate the reporting quality, methodological quality and evidence quality of these observational, cohort, and cross-sectional studies using Risk of Bias in Non-randomized Studies-of Environmental Exposure (ROBINS-E) assessment tool, and integrate the evidence within the GRADE framework. Additionally, the analysis of outcomes will be performed using the narrative synthesis and R software.

**Ethics and dissemination:**

Ethical approval is not required for this review. The results of this study will be disseminated through publication in a peer-reviewed journal.

**Study protocol registration:**

https://www.crd.york.ac.uk/PROSPERO/view/CRD420261281322, identifier (CRD420261281322).

## Introduction

Climate change has amplified the frequency and intensity of both individual and concurrent extreme weather and climate events, such as heat wave, cold spell, storms, heavy precipitation causing wildfires, floods, and droughts, which could adversely affect human health (1, 2). Recent observations indicate a significant increase in the frequency of extreme temperature events - heat wave and cold spell. Epidemiological evidence demonstrated that heat exposure significantly increases the risk of cardiovascular and respiratory diseases (3). In terms of cold exposure, global data from 2019 show that 2.92% of deaths and 1.03% of disability-adjusted life years (DALYs) were attributable to cold spell, with primary associated diseases being heart disease, stroke, and COPD (4). During the same period, heat exposure accounted for 0.54% of all-cause mortality and 0.46% of DALYs (5).

COPD is a heterogeneous pulmonary disease and ranks as the third leading cause of death worldwide, accounting for approximately 3.3 million deaths annually. The prevalence of COPD among adults aged 20 years and older in China is 8.6, and 13.7% for those aged 40 years and older (6). To this point, the majority of evidence substantiating the relationship between extreme temperature and the pathogenesis of COPD has stemmed from high - income countries. In these countries, studies have reported significant associations between high and low-temperature exposure and increased incidence and mortality rates of COPD. Data from Europe indicated that heat waves significantly elevate COPD mortality risk, with relative risks of 1.25 (95% CI: 1.09–1.43, *p* < 0.05) in Italy, and 1.26 (95% CI: 1.158–1.372, *p* < 0.05) in Spain (7, 8). A study from England reported that for every 1 °C increase in temperature during extreme heat events, the risk of COPD hospitalization rises by 1.47% (9). Compared with high temperature, low temperature appear to pose a greater health risk for COPD. A nationwide study in Spain found that the risk of cold-related hospitalization was 49.6% higher than that associated with high temperature (8), and a 1 °C decrease in temperature was associated with a 0.8% increase in the risk of COPD acute exacerbations (10). In recognition of these climate-related threats, the 2025 Global Initiative for Chronic Obstructive Lung Disease (GOLD) report introduced considerations of climate change for the first time, highlighting that patients with COPD face elevated mortality risks due to extreme temperature, with greater risks associated with colder environment. Furthermore, high temperature increase hospitalization risk, whereas low temperature contribute to greater disease severity in COPD patients (11).

China is a region highly sensitive to climate change and has experienced substantial impacts (12). In the context of the increasing frequency of extreme temperature events caused by climate change, it is of great significance to study the impact of extreme temperature on COPD patients in China. Such research is urgently needed to inform health policy development and to provide evidence for clinicians and public health professionals. The objective of this systematic review is to comprehensively assess the existing evidence regarding the relationship between extreme temperature exposure and COPD related health outcomes within the Chinese context. By aggregating research findings from multiple regions across China, the review aims to enhance the current understanding of how climate change, particularly the occurrence of extreme temperature events, is associated with the health burden of COPD in Chinese populations.

## Methods

### Study registration

This protocol has been officially registered with the PROSPERO platform under the registration number CRD420261281322. The initial registration took place on 8 January 2026. The research report will be prepared in strict accordance with the Preferred Reporting Items for Systematic Reviews and Meta-Analyses Protocol (PRISMA-P) 2015 statement (13). The complete PRISMA P 2015 checklist is provided as Supplementary file 1. The study began on 8 January 2026 and is anticipated to be finalized by 31 August 2026.

### Inclusion criteria

The PECOS research design framework will be used as the basis for determining the inclusion criteria of the primary studies (14).

### Population

This systematic review will focus on Chinese populations of varying regions with COPD health outcomes related to extreme temperature exposure.

### Exposure

This component of the study considers extreme temperature events. The temperature extremes will be defined using definitions presented by the included studies. For example, definitions based on percentiles versus those based on absolute thresholds.

### Comparison

This study will investigate the association between different levels of extreme temperature events (such as moderate and severe heat exposure) and all seasons of COPD in China.

### Outcomes

The study will evaluate the impact of extreme temperature on health outcomes, specifically focusing on COPD related mortality or morbidity including hospitalizations or acute exacerbations. Morbidity is defined as hospitalizations or emergency department visits with a primary diagnosis of COPD based on the ICD-10 code. We will distinguish emergency department visits that resulted in hospital admission (counted as hospitalizations) from those that do not (counted as emergency department visits only). To reduce potential misclassification with asthma or other respiratory diseases, records with a primary diagnosis of asthma or other chronic lower respiratory diseases will be excluded, unless COPD is coded as the primary diagnosis.

COPD-specific mortality, and COPD-related hospitalizations (defined as inpatient admissions with COPD as the primary diagnosis based on ICD-10 codes) will be selected as primary outcomes. The secondary outcomes will include COPD-related emergency department visits that do not result in hospitalization, and acute exacerbations of COPD (defined according to GOLD).

### Study design

The study design will incorporate observational studies (cohort, case–control, time-series, ecological time series, case-crossover, or case series study designs or cross-sectional designs) conducted in China. They provided effect estimates, such as relative risks or odds ratios, with confidence intervals, enabling a clear understanding of the associations between extreme temperature exposure and COPD health outcomes.

### Exclusion criteria

Studies meeting any of the following criteria will be excluded: (1) Case reports, opinion pieces, editorials or commentaries, unpublished studies, conference abstracts, or dissertations. (2) Global studies that did not provide separate data for Chinese population or studies involving non-China populations. (3) Studies included other respiratory diseases influenced by factors unrelated to extreme temperature, or those without relevant temperature exposure factors in the research. (4) Studies without outcome measures related to COPD or those unable to derive extractable effect estimates.

### Search strategy

The literature search will be conducted independently by two researchers (JX and PZ). In case of disagreement, a third researcher (YW) will participate in the discussion to reach a consensus. The literature will be searched from PubMed, Web of Science, Embase, CNKI, WANGFANG Data, VIP and Sinomed databases from inception to 8 January 2026. The retrieval words are a combination of subject words and free words. The search terms will include ‘Chronic Obstructive Pulmonary Disease’, ‘Pulmonary Disease, Chronic Obstructive’, ‘COPD’, ‘extreme temperature’, ‘extreme weather’ and others. The search strategy used in the PubMed database is provided in Table 1, and the complete search strategy is included in Supplementary file 2.

**Table 1:** Search strategy in PubMed.

No.	Search terms
#1	(“Chronic Obstructive Pulmonary Disease”[MeSH Terms] OR “Pulmonary Disease, Chronic Obstructive”[Text Word] OR COPD[Text Word] OR “emphysema”[Text Word])
#2	(“Weather”[MeSH Terms] OR “Climate Change”[MeSH Terms] OR “extreme weather”[Text Word] OR “heat wave”[Text Word] OR “cold spell”[Text Word] OR “extreme temperature”[Text Word] OR “high temperature”[Text Word] OR “low temperature”[Text Word] OR “temperature variability”[Text Word])
#3	(“China”[MeSH Terms] OR “Chinese counties”[Text Word])
#4	#1 AND #2 AND #3

### Study selection

**Figure 1:**
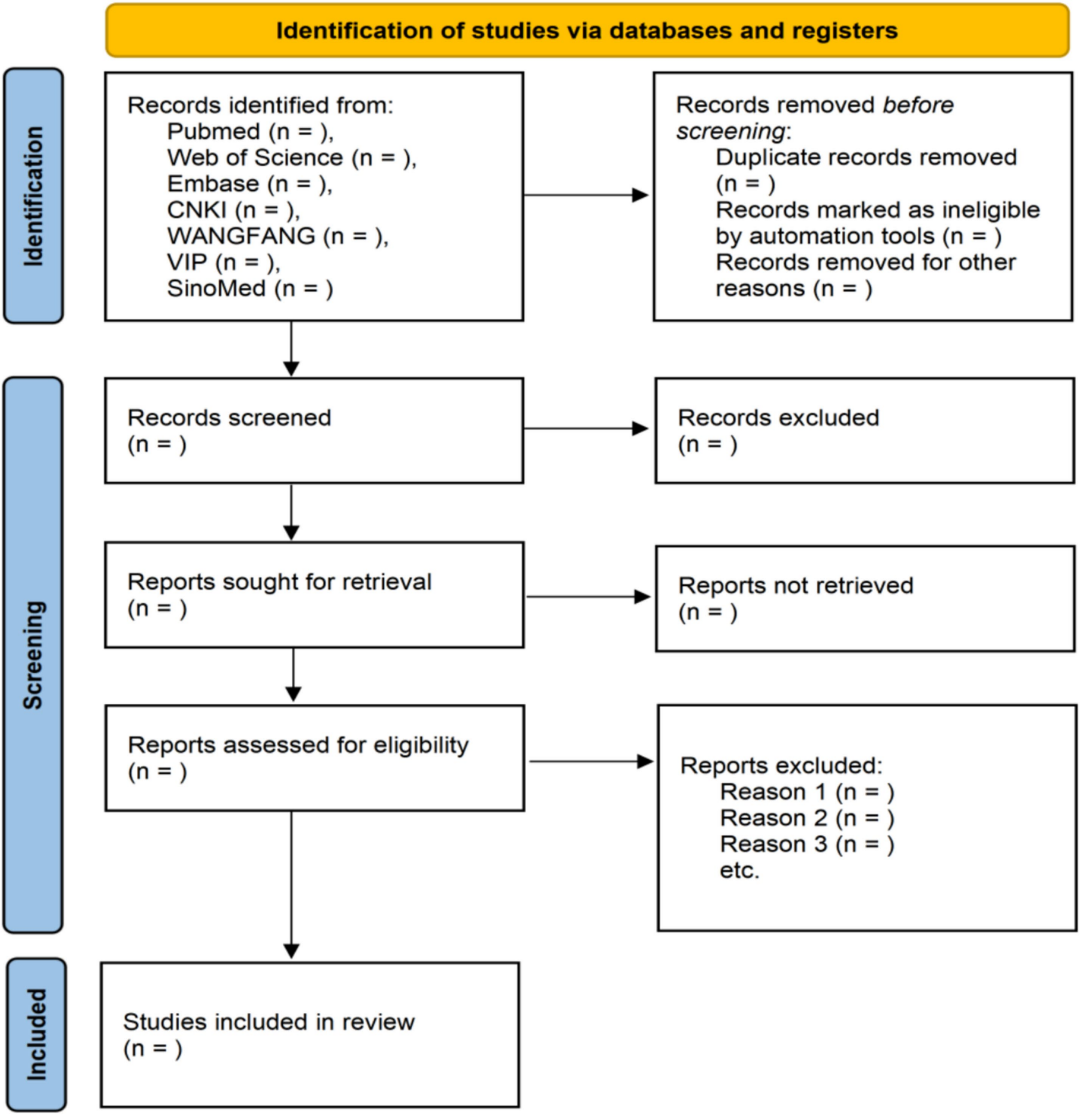
Flow chart diagram of study selection.

The NoteExpress software will be used to manage the full text review as well as the screening of titles and abstracts. Two independent reviewers (JX and YW) will assess the relevance of the publications based on titles and abstracts containing specific temperature related search phrases. Publications that pass the initial screening will then undergo evaluation against the predefined inclusion and exclusion criteria during the full text review. Any disagreements arising between the two reviewers will be resolved by consulting a third independent reviewer (SY). The complete study selection process is illustrated in Figure 1.

### Data extraction

The data extraction process will be carried out independently by two researchers (PZ and YW). The information gathered from studies that meet the eligibility criteria includes the author details, publication year, the study’s geographic location and its temporal scope, the total number of participants along with their sex distribution and age range, the main variable defining temperature exposure, and the principal outcomes. Whenever possible, additional characteristics will also be collected, such as the study region is coastal or inland areas.

### Quality assessment

The methodological quality and risk of bias of the included studies will be appraised by two independent reviewers (JX and JY) using the ROBINS-E tool, which is specifically designed for observational studies of environmental or occupational exposures (15). Inter-rater agreement between the two reviewers will be assessed at the title/abstract screening, full-text screening, and risk of bias assessment stages. Agreement will be quantified using Cohen’s kappa statistic to account for chance agreement, and 95% confidence intervals will be reported. If agreement is lower than 0.60, the two reviewers will discuss discrepancies and, if necessary, a third reviewer will be consulted to reach consensus. All calculations will be performed using SPSS version 26.0.

### Statistical analysis

To synthesis data for this systematic review, eligible studies will be organized by publication year, allowing a chronological assessment of evidence linking extreme temperature and COPD. Given anticipated heterogeneity in study designs, including exposure duration, statistical models, types of extreme temperature, and geographic locations within China, a narrative synthesis will be adopted. The synthesis will begin by summarizing key study characteristics, focusing on participant demographics (age, sex, disease course) and study design. Subsequently, the generalized association between extreme temperature exposure (high temperature, low temperature, heat wave, cold spell) and COPD outcomes will be described, followed by an independent analysis of temperature variability.

For the meta-analysis, we will assess statistical heterogeneity across studies using Cochran’s Q test and the I^2^ statistic (16). Given the anticipated substantial heterogeneity arising from diverse geographical regions, climatic conditions, exposure definitions, and study designs across China, a random-effects model will be applied *a priori* as the primary analysis for all pooled estimates (17). When the I^2^ < 40% and the Q test is not significant (*p* ≥ 0.1), indicating low statistical heterogeneity, we will additionally perform a sensitivity analysis using a fixed-effect model to evaluate the robustness of the pooled results. Pooled effect measures, including mean difference (MD) and risk ratio (RR) with 95% confidence intervals (CIs), will be calculated separately for each outcome and temperature exposure category. All statistical analyses will be conducted in R, and significance will be defined as *p* < 0.05. Additional details on the statistical methods are provided in the Supplementary file 3.

### Definition for extreme temperature

The World Health Organization (WHO) report defines a heat wave as a period characterized by abnormally hot days and nights over a certain duration, during which excessive heat accumulates in some areas (18). A global study further specifies a heat wave as a period lasting two or more days, during which both the daily minimum and maximum temperatures exceed the 95th percentile of the temperature observed between 1986 and 2005 (19). And the definition of a cold spell lasts at least 3 days by combining the threshold of the 5th percentile of the annual average daily temperature at a specific location (20). In China, a traditional heat wave is defined as three or more consecutive days with a daily maximum temperature ≥ 35 °C. The definition of cold spell refers to a region after the cold air transit, the daily minimum temperature decreased by 8 °C and above in 24 h, or decreased by 10 °C and above in 48 h, or decreased by 12 °C and above in 72 h, and the daily minimum temperature decreased to 4 °C or below (21). However, China has a vast territory and a large north–south span. Therefore, in this systematic review temperature extremes will be defined using definitions presented by the included studies.

## Discussion

COPD remains a major global health concern, with persistently high morbidity and mortality rates imposing a substantial disease burden. However, studies have reported that rising temperature can lead to a significant increase in the risk of acute exacerbations of COPD (9, 22), other studies have not found such a correlation (23, 24). In addition, studies have shown that the impact of cold exposure on the incidence and mortality of COPD vary among different geographical regions and populations (25). Another study also found a significant association between air pollution exposure and hospitalizations related to COPD at temperatures of approximately 0 °C and 25 °C (26). Given these inconsistencies, a systematic review and meta analysis is warranted to consolidate the existing evidence, resolve prior conflicting findings, and offer improved clarity regarding these associations.

Several systematic reviews and meta-analyses have investigated the effects of extreme temperature on respiratory health, including COPD. For instance, a global systematic review and meta-analysis identified a significant association between heatwaves and respiratory mortality, though the study focused primarily on heatwaves and did not specifically examine COPD morbidity or mortality (27). Some studies only conducted a systematic review without a meta-analysis, or the findings were geographically limited and not exclusively focused on COPD (28, 29). Collectively, the existing systematic review studies generally have the following significant limitation: insufficient special attention to COPD, lack of integration of quantitative research results, and limited research coverage in developing countries (especially China).

In this study, a comprehensive systematic review and meta-analysis will be carried out to systematically evaluate the impact of extreme temperature on the health outcomes of COPD population in China, which filled the research gap in this field in developing countries. At the same time, this study identifies vulnerable subgroups susceptible to extreme temperature by subgroup analysis based on variables such as age, gender, and region. By quantitatively synthesizing existing studies, this study provides new evidence-based evidence for understanding the disease burden of COPD in the context of climate change.

### Study period

This systematic review will be conducted from 8 January 2026 to 31 August 2026. Of this, the literature search will be completed by 30 April 2026 to ensure sufficient time for subsequent data extraction, quality evaluation, etc.

## References

[1] McMichael AJ Woodruff RE Hales S. Climate change and human health: present and future risks. Lancet. (2006) 367:859–69. doi: 10.1016/S0140-6736(06)68079-3, 16530580

[2] Watts N Amann M Arnell N Ayeb-Karlsson S Beagley J Belesova K . The 2020 report of the lancet countdown on health and climate change: responding to converging crises. Lancet. (2021) 397:129–70. doi: 10.1016/S0140-6736(20)32290-X 33278353 PMC7616803

[3] Zhang JD Cheng XF Min SH Guo RQ Wang RN He YT . Burden of non-communicable diseases attributable to high temperature in a changing climate from 1990 to 2019:a global analysis. BMC Public Health. (2024) 24:2475. doi: 10.1186/s12889-024-19947-z, 39261784 PMC11389303

[4] Liu J Li M Yang Z Liu D Xiao T Cheng J . Rising trend and regional disparities of the global burden of disease attributable to ambient low temperature, 1990-2019: An analysis of data from the global burden of disease 2019 study. J Glob Health. (2024) 14:04017. doi: 10.7189/jogh.14.04017, 38635810 PMC11026037

[5] Song J Pan R Yi W Wei Q Qin W Song S . Ambient high temperature exposure and global disease burden during 1990-2019: an analysis of the global burden of disease study 2019. Sci Total Environ. (2021) 787:147540. doi: 10.1016/j.scitotenv.2021.147540, 33992940

[6] Wang C Xu J Yang L Xu Y Zhang X Bai C . Prevalence and risk factors of chronic obstructive pulmonary disease in China (the China pulmonary health [CPH] study): a national cross-sectional study. Lancet. (2018) 391:1706–17. doi: 10.1016/S0140-6736(18)30841-9, 29650248

[7] Oudin Åström D Schifano P Asta F Lallo A Michelozzi P Rocklöv J . The effect of heat waves on mortality in susceptible groups: a cohort study of a mediterranean and a northern European City. Environ Health. (2015) 14:30. doi: 10.1186/s12940-015-0012-0, 25889290 PMC4397690

[8] Achebak H Rey G Lloyd SJ Quijal-Zamorano M Méndez-Turrubiates RF Ballester J. Ambient temperature and risk of cardiovascular and respiratory adverse health outcomes: a nationwide cross-sectional study from Spain. Eur J Prev Cardiol. (2024) 31:1080–9. doi: 10.1093/eurjpc/zwae021, 38364198

[9] Konstantinoudis G Minelli C Vicedo-Cabrera AM Ballester J Gasparrini A Blangiardo M. Ambient heat exposure and COPD hospitalisations in England: a nationwide case-crossover study during 2007-2018. Thorax. (2022) 77:1098–104. doi: 10.1136/thoraxjnl-2021-218374, 35459745 PMC9606528

[10] Tseng CM Chen YT Ou SM Hsiao YH Li SY Wang SJ . The effect of cold temperature on increased exacerbation of chronic obstructive pulmonary disease:a nationwide study. PLoS One. (2013) 8:e57066. doi: 10.1371/journal.pone.0057066, 23554858 PMC3598847

[11] Global Initiative for Chronic Obstructive Lung Disease. Global strategy for the diagnosis, management, and prevention of chronic obstructive pulmonary disease: 2025 report [EB/OL]. (2024). Available at: https://goldcopd.org/2025-gold-report/

[12] Yin Z Zhou B Duan M Chen H Wang H. Climate extremes become increasingly fierce in China. Innovation. (2023) 4:100406. doi: 10.1016/j.xinn.2023.100406, 36910135 PMC9999194

[13] Moher D Shamseer L Clarke M Ghersi D Liberati A Petticrew M . Preferred reporting items for systematic review and meta-analysis protocols (PRISMA-P) 2015 statement. Syst Rev. (2015) 4:1. doi: 10.1186/2046-4053-4-1, 25554246 PMC4320440

[14] Morgan RL Whaley P Thayer KA Schünemann HJ. Identifying the PECO: a framework for formulating good questions to explore the association of environmental and other exposures with health outcomes. Environ Int. (2018) 121:1027–31. doi: 10.1016/j.envint.2018.07.015, 30166065 PMC6908441

[15] Morgan RL Thayer KA Santesso N Holloway AC Blain R Eftim SE . A risk of bias instrument for non-randomized studies of exposures: a users' guide to its application in the context of GRADE. Environ Int. (2019) 122:168–84. doi: 10.1016/j.envint.2018.11.004, 30473382 PMC8221004

[16] Higgins JPT Thompson SG Deeks JJ Altman DG. Measuring inconsistency in meta-analyses. BMJ. (2003) 327:557–60. doi: 10.1136/bmj.327.7414.557, 12958120 PMC192859

[17] Xiao Q Xue B Huang Y Wang M. Effectiveness of biologics for patients with severe asthma: study protocol for an umbrella review of systematic reviews and meta-analyses. BMJ Open. (2025) 15:e096874. doi: 10.1136/bmjopen-2024-096874, 40254309 PMC12010295

[18] World Health Organization. Heat and Health [EB/OL]. (2024). Available at: https://www.who.int/zh/news-room/fact-sheets/detail/climate-change-heat-and-health

[19] Romanello M Napoli CD Green C Kennard H Lampard P Scamman D . The 2023 report of the lancet countdown on health and climate change: the imperative for a health-centred response in a world facing irreversible harms. Lancet. (2023) 402:2346–94. doi: 10.1016/S0140-6736(23)01859-7, 37977174 PMC7616810

[20] Gao Y Huang W Zhao Q Ryti N Armstrong B Gasparrini A . Global, regional, and national burden of mortality associated with cold spells during 2000-19: a three-stage modelling study. Lancet Planet Health. (2024) 8:e108–16. doi: 10.1016/S2542-5196(23)00277-2, 38331527

[21] Zou Z. Association between Extreme Temperature Events, Atmospheric Pollutants, and Cardiovascular Disease Mortality. Guangzhou City, China: Southern Medical University (2025).

[22] Rai M Breitner S Huber V Zhang S Peters A Schneider A. Temporal variation in the association between temperature and cause-specific mortality in 15 German cities. Environ Res. (2023) 229:115668. doi: 10.1016/j.envres.2023.115668 36958378

[23] Han J Liu S Zhang J Zhou L Fang Q Zhang J . The impact of temperature extremes on mortality: a time-series study in Jinan, China. BMJ Open. (2017) 7:e014741. doi: 10.1136/bmjopen-2016-014741, 28465307 PMC5566622

[24] Zhang Y Liu X Kong D Fu J Liu Y Zhao Y . Effects of ambient temperature on acute exacerbations of chronic obstructive pulmonary disease: results from a time-series analysis of 143318 hospitalizations. Int J Chron Obstruct Pulmon Dis. (2020) 15:213–23. doi: 10.2147/COPD.S224198, 32099346 PMC6996111

[25] Rau A Tarr GAM Baldomero AK Wendt CH Alexander BH Berman JD. Heat and cold wave-related mortality risk among United States veterans with chronic obstructive pulmonary disease: a case-crossover study. Environ Health Perspect. (2024) 132:27004. doi: 10.1289/EHP13176, 38334741 PMC10855215

[26] Birinci E Çeker AO Çapraz Ö Özdemir H Deniz A. Lagged and temperature-dependent effects of ambient air pollution on COPD hospitalizations in Istanbul. Environments (Basel). (2026) 13:56. doi: 10.3390/environments13010056

[27] Cheng J Xu Z Bambrick H Prescott V Wang N Zhang Y . Cardiorespiratory effects of heatwaves: a systematic review and meta-analysis of global epidemiological evidence. Environ Res. (2019) 177:108610. doi: 10.1016/j.envres.2019.108610, 31376629

[28] Witt C Schubert AJ Jehn M Holzgreve A Liebers U Endlicher W . The effects of climate change on patients with chronic lung disease. A systematic literature review. Dtsch Arztebl Int. (2015) 112:878–83. doi: 10.3238/arztebl.2015.0878, 26900154 PMC4736555

[29] Hadei M Hopke PK Aghababaeian H Faridi S Hasham Firooz M Ostadtaghizadeh A. Association of heat and cold waves with cause-specific mortality in Iran: a systematic review and meta-analysis. Sci Rep. (2024) 14:23327. doi: 10.1038/s41598-024-74821-7, 39375490 PMC11458800

