# Supplementary material for: Association between extreme temperature exposure and COPD health outcomes in China: study protocol for a systematic review
Source: Front Med (Lausanne). 2026 Jun 25;13:1873054. doi: 10.3389/fmed.2026.1873054 (PMC13346046; doi:10.3389/fmed.2026.1873054)
Supplement: Supplementary file 2 [file Data_Sheet_2.DOCX]

**Supplementary file 2.** The details of the search strategy.

| **Pubmed** |
| --- |
| #1 ("Chronic Obstructive Pulmonary Disease"[MeSH Terms] OR "Pulmonary Disease, Chronic Obstructive"[Text Word] OR "COPD"[Text Word] OR "emphysema"[Text Word])  #2 ("Weather"[MeSH Terms] OR "Climate Change"[MeSH Terms] OR "extreme weather"[Text Word] OR "heat wave"[Text Word] OR "cold spell"[Text Word] OR "extreme temperature"[Text Word] OR "high temperature"[Text Word] OR "low temperature"[Text Word] OR "temperature variability"[Text Word])  #3 ("China"[MeSH Terms] OR "Chinese counties"[Text Word])  #4 #1 AND #2 AND #3 |
| **Embase** |
| #1 ('copd'/exp OR copd OR 'chronic obstructive pulmonary disease'/exp OR 'chronic obstructive pulmonary disease' OR 'emphysema'/exp OR emphysema)  #2 ('extreme' AND 'weather' OR 'climate change' OR 'heat wave' OR 'cold spell' OR 'extreme temperature' OR 'high temperature' OR 'low temperature' OR 'temperature variability')  #3 ('China' OR 'Chinese counties')  #1 AND #2 AND #3 |
| **Web of Science** |
| TS=("Chronic Obstructive Pulmonary Disease" OR "COPD" OR "emphysema") AND TS=("extreme weather" or "heat wave" or "cold spell" or "extreme temperature" or "high temperature" or "low temperature") AND TS=("China" or "Chinese counties") |
| **Chinese National Knowledge Infrastructure (CNKI)** |
| #1 TKA=慢性阻塞性肺疾病 + 慢阻肺 + COPD + copd  #2 TKA=极端温度 + 极端温度事件 + 极端温度环境 + 极端温度气候事件 + 极端温度变化 + 极端气候 + 极端气候变化 + 极端气候事件 + 热浪 + 冷空气 + 高温 +低温  #3 #1 AND #2 |
| **WANGFANG Data** |
| #1 题名或关键词:(慢性阻塞性肺疾病 OR 慢阻肺 OR COPD OR copd )  #2 题名或关键词:(极端温度 OR 极端温度事件 OR 极端温度环境 OR 极端温度气候事件 OR 极端温度变化 OR 极端气候 OR 极端气候变化 OR 极端气候事件 OR 热浪 OR 冷空气 OR 高温 OR 低温)  #3 #1 AND #2 |
| **VIP** |
| (M=慢性阻塞性肺疾病 OR 慢阻肺 OR COPD OR copd) AND (M=极端温度 OR 极端温度事件 OR 极端温度环境 OR 极端温度气候事件 OR 极端温度变化 OR 极端气候 OR 极端气候变化 OR 极端气候事件 OR 热浪 OR 冷空气 OR 高温 OR低温) |
| **Sinomed** |
| #1 ( "慢性阻塞性肺疾病"[常用字段:智能] OR "慢阻肺"[常用字段:智能] OR "COPD"[常用字段:智能] OR "copd"[常用字段:智能])  #2 ( "极端温度"[常用字段:智能] OR "极端温度事件"[常用字段:智能] OR "极端温度环境"[常用字段:智能] OR "极端温度气候事件"[常用字段:智能] OR "极端温度变化"[常用字段:智能] OR "极端气候"[常用字段:智能] OR "极端气候变化"[常用字段:智能] OR "极端气候事件"[常用字段:智能] OR "热浪"[常用字段:智能] OR "冷空气"[常用字段:智能] OR "高温"[常用字段:智能] OR "低温"[常用字段:智能])  #3 #1 AND #2 |
