# Supplementary material for: Association between extreme temperature exposure and COPD health outcomes in China: study protocol for a systematic review
Source: Front Med (Lausanne). 2026 Jun 25;13:1873054. doi: 10.3389/fmed.2026.1873054 (PMC13346046; doi:10.3389/fmed.2026.1873054)
Supplement: Supplementary file 3 [file Data_Sheet_3.DOCX]

**Supplementary file 3. Supplemented Statistical Analysis Plan**.

(1) Primary analysis restricted to comparable designs.

The core quantitative synthesis will focus on time-series and case-crossover studies, as they are the most commonly used designs in temperature–health research and typically report relative risk (RR) derived from distributed lag non-linear models. These designs share a common estimand (short-term, population-averaged RR) and can be synthesized with acceptable heterogeneity.

(2) Stratification by study design in subgroup analyses.

When other designs are included, they will be analyzed in separate subgroups: (a) time-series and case-crossover studies; (b) ecological time-series studies; (c) cohort studies (e.g., reporting hazard ratios or time-stratified RR); and (d) case-control and cross-sectional studies (typically reporting odds ratios). Quantitative pooling will be performed only within each design stratum where estimates are comparable. If a stratum contains too few studies, results will be summarized narratively.

(3) Handling different effect metrics.

For case-control and cross-sectional studies reporting odds ratios (OR), we will evaluate whether the outcome is sufficiently rare (e.g., mortality) to safely approximate OR as RR. If this condition is met, studies may be included alongside RR-based estimates, with a sensitivity analysis excluding OR-based studies to test robustness. When the rare-outcome assumption is not tenable (e.g., morbidity outcomes with high prevalence), OR-based studies will be described narratively and excluded from the quantitative synthesis.

(4) Addressing cumulative versus lag-specific effects.

To address cumulative versus lag-specific effects, we will group studies by their reported lag structure (e.g., single-day lag, cumulative lag over 0–21 days, or overall cumulative association) and perform separate meta-analyses within each lag category. When lag definitions are inconsistent across studies and prevent meaningful grouping, we will refrain from quantitative pooling and instead provide a structured narrative synthesis.

(5) Sensitivity analyses according to the design.

To assess the influence of study design on the pooled estimates, we will conduct sensitivity analyses by sequentially excluding each design type and comparing the results. Additionally, we will evaluate whether design-related heterogeneity is captured by the risk of bias assessment and, if feasible, restrict the primary synthesis to studies with low risk of bias.

(6)Subgroup Analysis

1) Percentile-based definitions (e.g., temperature exceeding the 95th percentile of a historical reference period); 2) Absolute threshold-based definitions (e.g., daily maximum temperature ≥ 35°C); 3) Official meteorological standards (e.g., the China Meteorological Administration definitions for heat waves and cold spells). If a sufficient number of studies permit, these subgroups will be analyzed separately. In addition, we will conduct sensitivity analyses by excluding studies with non-standard or ambiguous definitions to assess the robustness of the pooled estimates. When heterogeneity remains substantial within subgroups, we will refrain from quantitative synthesis and instead present a narrative summary.

Subgroup analyses will also be performed according to: 1) age group: The age groups in the study will be categorized as <60 or 65 or 75 years, 60–75 years, and ≥60 or 65 or 75 years based on similar research; 2) sex group: The sex groups will be categorized as male or female; 3) geographical region (e.g., northern vs. southern China, coastal vs. inland areas, and climate zones).
